# Quantitative analysis of da Vinci 5 force feedback technology in robotic rectal surgery: a preliminary pilot study

**DOI:** 10.1007/s00464-026-12842-1

**Published:** 2026-05-28

**Authors:** Yusuke Nishi, Yasumitsu Hirano, Yasuhiro Ishiyama

**Affiliations:** Department of Gastroenterological Surgery, Saitama Medical University International Medical Center, 1397-1 Yamane, Hidaka, Saitama 350-1298 Japan

**Keywords:** Robotic surgery, Da Vinci 5, Force Feedback, Colorectal surgery, Haptics, D3 dissection

## Abstract

**Background:**

The da Vinci 5 (DV5) introduces force feedback (FFb) technology to robotic surgery, aiming to enhance safety through haptic sensation. However, its quantitative impact on specific tissue manipulations in rectal cancer surgery remains unclear. This study evaluated the clinical significance of FFb by analyzing force metrics across different instrument roles.

**Methods:**

We retrospectively analyzed 13 consecutive robotic rectal cancer resections performed by a single expert surgeon. Force data were extracted from system logs for the Cadiere Forceps (static retraction) and Fenestrated Bipolar Forceps (dynamic retraction). Comparisons were made between three settings: Off (*N* = 3), Low (*N* = 11), and Medium (*N* = 5). For "Low" and "Medium" settings, cases overlapped as settings were adjusted intraoperatively. Cumulative force usage time was calculated for each setting. Statistical significance was assessed using the Kruskal–Wallis test.

**Results:**

A total of 91,000 + seconds for Cadiere and 68,000 + seconds for Fenestrated Bipolar forceps were analyzed. For the Cadiere Forceps (static retraction), the mean force was significantly reduced as FFb sensitivity increased (Off: 3.07 N, Low: 2.58 N, Medium: 2.03 N; *P* = 0.039). For the Fenestrated Bipolar Forceps (dynamic retraction), while the mean force showed no significant difference, the median maximum (peak) force was significantly suppressed with higher FFb settings (Off: 36.19 N, Low: 18.82 N, Medium: 10.06 N; *P* = 0.033). No intraoperative complications related to tissue trauma occurred.

**Conclusions:**

FFb technology in the DV5 effectively modulates surgical force based on the functional role of the instrument. It significantly reduces sustained stress during static retraction and serves as a "safety brake" to cap peak forces during dynamic maneuvers, potentially enhancing the safety of robotic rectal surgery.

Robotic-assisted surgery (RAS) has established itself as a standard approach in colorectal surgery, particularly for rectal cancer, due to its high-definition 3D visualization and multi-articulated instrumentation [1]. However, a major limitation of conventional platforms (e.g., da Vinci Xi) has been the lack of haptic feedback. Surgeons have historically relied on "visual haptics"—estimating force based on tissue deformation. This compensatory mechanism is highly dependent on surgeon experience and carries risks of unintended tissue damage or suture breakage, especially during off-screen manipulation or when handling rigid tissues [2].

In July 2025, the fifth-generation system, "da Vinci 5," was introduced in Japan. This system is equipped with force feedback (FFb) technology, which detects force directly via strain gages embedded in the instrument tips and provides physical resistance to the surgeon through the console hand controllers [3].

Early reports from the United States have been promising, suggesting FFb usage reduces the average force applied to tissues in thoracic and general surgery [4, 5]. However, detailed quantitative analyses regarding the impact of FFb in colorectal surgery—a field requiring diverse manipulations such as D3 dissection and mesenteric elevation—remain limited [6].

The objective of this pilot study is to report our preliminary clinical experience with the da Vinci 5 and to quantitatively clarify how FFb modulation occurs during distinct surgical maneuvers.

## Material and methods

### Patients and study design

This pilot study included 13 consecutive patients with rectal cancer who underwent robotic resection using the da Vinci 5 system at Saitama Medical University International Medical Center between July 2025 and November 2025. To minimize procedural variability and allow for a precise comparison, all cases were performed by a single expert surgeon (Y.H.) who is board-certified and qualified as a robotic surgery proctor. This retrospective study was approved by the Institutional Review Board of Saitama Medical University International Medical Center (IRB Protocol No. I2026-046). The requirement for informed consent was waived, and an opt-out approach was used.

## Working definitions of tissue manipulation and surgical instruments

**Fig. 1:**
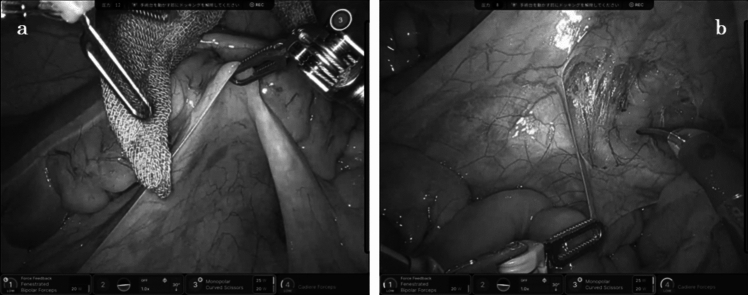
Comparison of tissue manipulation strategies. **a** Static retraction (left image): the Cadiere forceps (4th arm) is used for simple elevation of the mesocolon, including the inferior mesenteric artery (IMA). This role requires maintaining a static field without excessive force. **b** Dynamic retraction (right image): the fenestrated bipolar forceps (1st arm) is used for strong traction on the peritoneum. This applies tension to the dissection plane to facilitate cutting, requiring "necessary force" even with force feedback active

To analyze the impact of FFb in different clinical contexts, we employed the following working definitions (Fig. 1):

1. Static Retraction: Steady, constant elevation of the mesocolon or vessels (e.g., IMA) using the Cadiere Forceps (4th arm) to maintain the surgical field exposure.
2. Dynamic Retraction: Active counter-traction applied by the Fenestrated Bipolar Forceps (1st arm) to create appropriate tension on the dissection plane during D3 lymphadenectomy.

## FFb settings and data extraction

The da Vinci 5 FFb function allows for "Off," "Low," "Medium," and "High" settings. In this study, we compared three levels: "Off" (baseline), "Low" (standard), and "Medium" (enhanced). The "High" setting was intentionally avoided because the physical feedback was perceived as excessively strong, potentially hindering smooth traction. For the "Off" group, the first three consecutive cases were analyzed after excluding entries with zero usage time to ensure a robust baseline.

The choice of FFb setting was determined on a case-by-case basis at the surgeon’s discretion. While "Low" was frequently used as the initial setting, in some procedures, the "Medium" setting was pre-selected from the commencement of the surgery to evaluate its continuous impact throughout various surgical phases. This approach allowed the surgeon to assess the feasibility and haptic utility of different sensitivity levels across a broad range of maneuvers. Consequently, the settings were not necessarily escalated sequentially but were utilized as independent tools to optimize haptic perception based on the surgeon’s intraoperative goals.

Quantitative force data were prospectively extracted from system logs, focusing on: (1) Mean Force (N), (2) Max Force (N), and (3) Cumulative Duration (seconds) for each setting.

## Statistical analysis

Statistical analyses were conducted using Python (version 3.10) with the SciPy (version 1.10.1) and pandas (version 2.0.3) libraries. Continuous variables are expressed as mean ± standard deviation or median with interquartile range (IQR), depending on the data distribution. Given the small sample size of the baseline group and the non-normal distribution of the data, the Kruskal–Wallis test was employed to compare force metrics across the three FFb settings (Off, Low, and Medium). Statistical significance was defined as *P* < 0.05. As certain FFb settings were adjusted intraoperatively within the same procedure, resulting in a partial overlap of cases across groups, the Kruskal–Wallis test was utilized as an exploratory nonparametric comparison. Consequently, these results should be interpreted with caution.

## Results

### Clinical outcomes

The median age of the 13 patients was 68.2 years (range: 39–88). All procedures were successfully completed robotically with zero conversions to open or laparoscopic surgery. The median operative time was 254.8 min, and the median estimated blood loss was 41.8 mL. Postoperative complications occurred in 1 case (7.7%), which was a postoperative ileus classified as Clavien-Dindo grade IIIa. This was managed with radiologic intervention and was determined to be unrelated to the use of the da Vinci 5 system or the FFb function. No device-related adverse events were observed (Table 1).

**Table 1:** Patient demographics and perioperative outcomes (*n* = 13)

Variable	Value (*n* = 13)
Age (year)	68.2(39–88)
Sex (male/female)	11/2
BMI (Kg/m^2^)	21.66(17.5–27.0)
Tumor location	RS:1, Ra:2, Rb:10
Operative outcomes	
Operative time (min)	254.8 (171–471)
Estimated blood loss (mL)	41.8 (5–210)
Conversion to lap/open	0 (0%)
Postoperative outcomes	
Length of stay (days)	10.8 (8–18)
Complications (CD ≥ IIIa)	1(7.7%) (Ileus: 1)

## Quantitative force analysis by instrument role

**Fig. 2:**
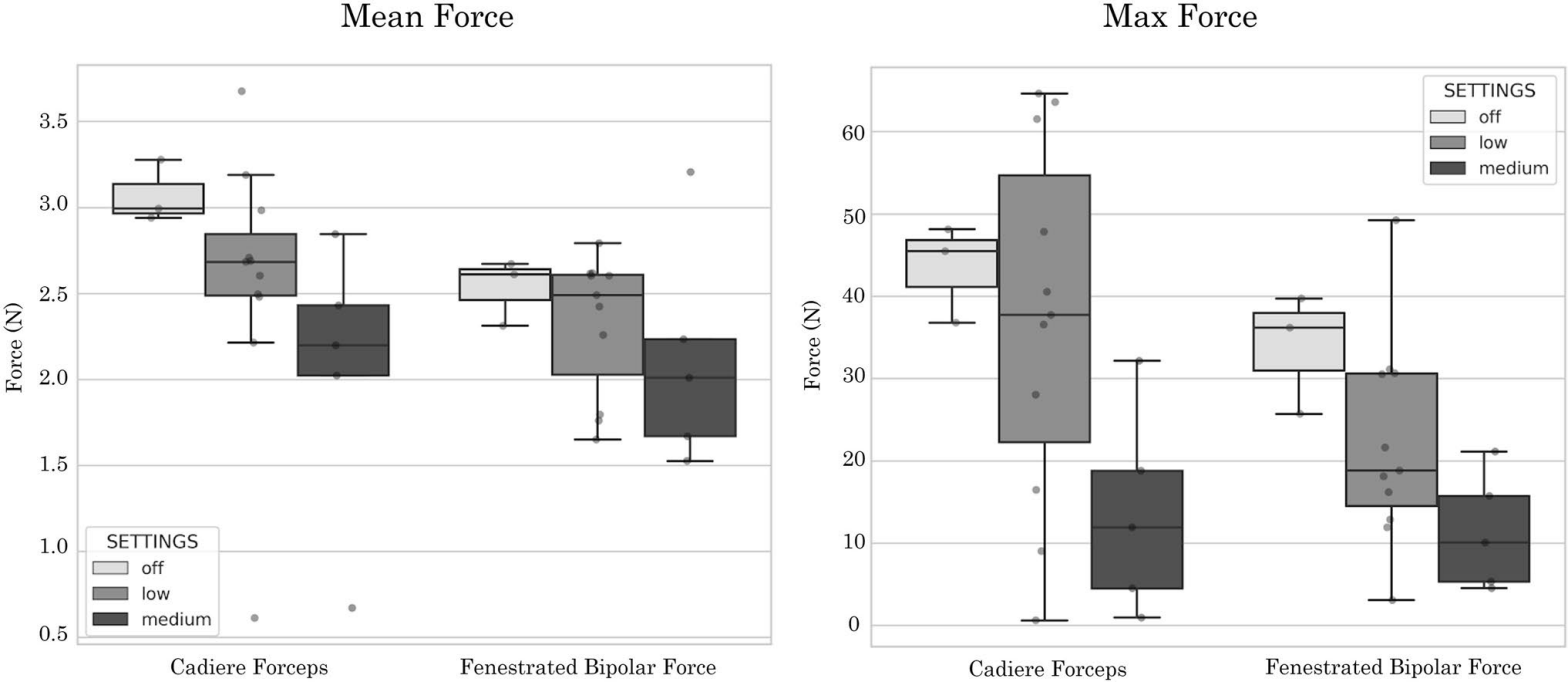
Quantitative assessment of surgical force across different force feedback (FFb) settings. (Left) Mean force: Comparison of the average force applied by the Cadiere forceps (static retraction) and fenestrated bipolar forceps (dynamic retraction) across different FFb levels. (Right) Max force: Comparison of the maximum peak force recorded. Each box plot represents the median and interquartile range (IQR), with individual dots representing raw data from each surgical case. Note the consistent reduction in both mean and peak forces as FFb sensitivity is increased

The quantitative analysis of force metrics, summarized in Table 2 and Fig. 2, was based on a vast dataset exceeding 91,000 s of cumulative usage for the Cadiere Forceps and 68,000 s for the Fenestrated Bipolar Forceps.

**Table 2:** Quantitative force analysis by instrument role and force feedback (FFb) Setting

Instrument (Role)	FFb Setting	N (Cases)	Cumulative Time (sec)	Mean Force, N (SD)	Median Max Force, N [IQR]	P-value
Cadiere forceps (static retraction)	Off	3	18,615	3.07 (0.18)	45.48 [5.68]	(Mean) 0.039*
	Low	11	61,846	2.58 (0.76)	37.74 [32.44]	(Max) 0.067‡
	Medium	5	10,542	2.03 (0.82)	11.92 [14.29]	
Fenestrated bipolar forceps (dynamic retraction)	Off	3	14,732	2.53 (0.19)	36.19 [7.01]	Mean) 0.311
	Low	11	46,738	2.33 (0.40)	18.82 [16.07]	(Max) 0.033**
	Medium	5	7,351	2.13 (0.66)	10.06 [10.41]	

*SD* Standard Deviation, *IQR* Interquartile Range.† N (Cases): Total number of unique surgical cases in which the specific FFb setting was utilized

Note that for "Low" and "Medium" settings, the same cases overlap because settings were adjusted intraoperatively based on the specific surgical task. "Off" represents the initial baseline cases

^*^P = 0.039$ for Mean Force (Kruskal–Wallis test across Off, Low, and Medium settings)

^**^P = 0.033$ for Max Force (Kruskal–Wallis test across Off, Low, and Medium settings)

^‡^*P* = 0.067$ for Max Force (Kruskal–Wallis test), indicating a strong trend toward reduction

For the Cadiere Forceps (static retraction), there was a statistically significant reduction in mean force as the FFb setting was increased (Off: 3.07 ± 0.18 N; Low: 2.58 ± 0.76 N; Medium: 2.03 ± 0.82 N; *p* = 0.039). The median maximum force also showed a strong downward trend from 45.48 N (Off) to 11.92 N (Medium) (*p* = 0.067).

Regarding the Fenestrated Bipolar Forceps (dynamic retraction), while the mean force did not differ significantly, the median maximum (peak) force was significantly suppressed with higher FFb settings (Off: 36.19 N; Low: 18.82 N; Medium: 10.06 N; *p* = 0.033).

It should be noted that for the "Low" and "Medium" settings, the case numbers overlap because the sensitivity was adjusted intraoperatively according to the specific surgical maneuver.

## Discussion

This study is among the first to quantitatively demonstrate that the da Vinci 5’s FFb technology does not uniformly dampen surgical force but rather exercises "selective intervention" depending on the context of the surgical maneuver.

## Clinical implications of force feedback settings

Our quantitative findings demonstrate that the FFb technology of the da Vinci 5 effectively modulates surgical force according to the instrument’s role. In static retraction (Cadiere Forceps), where constant elevation of the mesocolon is required, the FFb "Medium" setting significantly lowered the average force applied, potentially reducing the risk of prolonged ischemic or mechanical stress to the tissues.

In contrast, dynamic maneuvers (Fenestrated Bipolar Forceps) often necessitate a "necessary force" to maintain tension on the dissection plane. Our data revealed that even in these scenarios, activating FFb significantly capped the peak forces (Max Force). The reduction of median peak force from 36.19 N to 10.06 N suggests that FFb acts as a "safety brake," preventing accidental excessive traction that could lead to tissue tearing.

Although the number of baseline cases (Off) was limited, the analysis of tens of thousands of seconds of surgical data provides a robust quantitative basis for the safety and utility of FFb in rectal cancer surgery.

## Limitations

Several limitations of this study should be acknowledged. First, it is a retrospective, single-center pilot study with a small sample size (*n* = 13). Although we performed exploratory nonparametric statistical testing, the results should be interpreted with caution due to the limited cohort and the potential for Type II errors.

Second, the comparison between the FFb "Off" and "On" settings was chronological, which may introduce a learning curve bias as the surgeon gained more experience with the da Vinci 5 system during the study period.

Third, because FFb settings were adjusted intraoperatively based on the surgical task, there was a partial overlap of cases between the "Low" and "Medium" groups. This lack of complete independence between groups means the results should be viewed as exploratory trends.

Fourth, this series was conducted by a single expert surgeon, and the findings may not be directly generalizable to surgeons at different stages of their robotic surgery training.

Finally, we focused on quantitative force metrics as surrogate markers for safety. While higher FFb settings, particularly the "Medium" setting, effectively reduced both mean and peak forces, the direct correlation between these reductions and clinical outcomes, such as anastomotic leakage or long-term tissue viability, remains to be established in larger prospective trials.

## Conclusion

The force feedback (FFb) technology of the da Vinci 5 was safely integrated into robotic rectal cancer surgery. Our preliminary quantitative analysis suggests that FFb facilitates task-specific force modulation: it effectively reduces sustained force during static retraction (4th arm) while allowing the surgeon to maintain the tension necessary for dynamic maneuvers (1st arm). Although these findings are based on a small pilot cohort, they indicate that real-time haptic feedback supports surgeons in achieving more precise force regulation. Future prospective studies are required to validate these findings and correlate force reduction with long-term clinical outcomes.
